# Probiotics (*Bacillus clausii* and *Lactobacillus fermentum* NMCC-14) Ameliorate Stress Behavior in Mice by Increasing Monoamine Levels and mRNA Expression of Dopamine Receptors (D_1_ and D_2_) and Synaptophysin

**DOI:** 10.3389/fphar.2022.915595

**Published:** 2022-07-19

**Authors:** Mujeeb Ur Rehman, Shakira Ghazanfar, Rizwan Ul Haq, Shakir Ullah, Salman Khan, Jianbo Wu, Waqar Ahmad, Muhammad Khalid Tipu

**Affiliations:** ^1^ Department of Pharmacy, Faculty of Biological Sciences, Quaid-i-Azam University, Islamabad, Pakistan; ^2^ National Institute for Genomics and Advanced Biotechnology (NIGAB), National Agricultural Research Centre (NARC), Islamabad, Pakistan; ^3^ Department of Pharmacy, Abbottabad University of Science & Technology, Abbottabad KPK, Pakistan; ^4^ Institute of Basic Medical Sciences, Khyber Medical University, Peshawar KPK, Pakistan; ^5^ Collaborative Innovation Center for Prevention and Treatment of Cardiovascular Diseases of Sichuan Province, Drug Discovery and Functional Food Laboratory, Southwest Medical University, Luzhou, China; ^6^ Laboratory for Cardiovascular Pharmacology, Department of Pharmacology, School of Pharmacy, Southwest Medical University, Luzhou, China

**Keywords:** *Bacillus clausii*, gut–brain axis, *Lactobacillus fermentum* NMCC-14, neurological disorders, probiotics, restraint stress

## Abstract

Stress is a physiological consequence of the body to adversity. The gut–brain axis and probiotics are gaining interest to provide better treatment for stress and other neurological disorders. Probiotic (*Lactobacillus fermentum* NMCC-14 and *Bacillus clausii*, 10^10^ colony-forming unit/day/animal, per oral) effects were investigated in acute (up to day 7) and subacute (days 8–14) restraint-stressed and normal mice through behavioral paradigms (elevated plus maze: EPM, light dark box/dark light box: LDB, and open field test: OFT). Time spent in the open arms of the EPM, time spent in the light compartment of the LDB, and movable time and time spent in the center of the OFT were significantly (*p* ≤ 0.05, *n* = 5) increased in probiotic-treated restraint-stressed mice. Enzyme-linked immunoassay determined blood cortisol and adrenocorticotropic hormone (ACTH) levels, which were reduced significantly (*p* < 0.05, *n* = 5) in probiotic-treated restraint-stressed mice. Hematoxylin and eosin-stained hippocampal slides also showed less or no neurodegeneration in the probiotic-treated animals. High-performance liquid chromatography and quantitative polymerase chain reaction were performed to determine the monoamine levels and mRNA expression of dopamine receptor subtypes (D_1_ and D_2_) and synaptophysin in the mice hippocampus (HC) and prefrontal cortex (PFC). The dopamine, serotonin, and norepinephrine levels were also significantly (*p* < 0.05, *n* = 5) increased in the HC and PFC of probiotic-treated animal brains. Fold expression of mRNA of D_1_ and D_2_ (except HC, LF-S, day 14) receptors and synaptophysin was also significantly (*p* < 0.05, *n* = 5) increased in the same brain parts of probiotic-treated restraint-stressed mice. Comparing mice in the *Lactobacillus fermentum* NMCC-14 and *Bacillus clausii* groups to mice in the normal group, only a significant (*p* < 0.05, *n* = 5) decrease was observed in the serum ACTH and cortisol levels on day 14 in *Bacillus clausii*-treated mice, where all other parameters also showed improvement. In comparison, *Bacillus clausii* showed greater stress suppressant activity than *Lactobacillus fermentum* NMCC-14. However, both probiotic bacteria can be a better and safer therapeutic alternative for ailments than currently available drugs.

## 1 Introduction

Stress is a non-specific response and physiological consequence of the body to adversity with the feeling of emotional or physical tension (Habib et al., 2001; Tan and Yip, 2018). It originates from any life’s event or thinking that affects emotions or mood of an individual to be nervous and angry, or frustrated. In a short burst, stress can be positive to avoid any danger or confront everyday challenges. If the symptoms are recurrent with moderate or intense severity and persistent enough to produce long-lasting feelings, this can promote, mediate, or even cause serious mental health conditions and psychopathologies like post-traumatic stress disorder, depression, schizophrenia, multiple sclerosis, Alzheimer’s disease, dementia, and memory impairment (Esch et al., 2002; Van Praag, 2004; Shansky et al., 2006).

Any exogenous stimuli or alteration may initiate a stress response in endogenous homeostasis. These changes activate the sympathetic division and the hypothalamic–pituitary–adrenal (HPA) axis (Feodorova and Sarafian, 2012). Densely packed neurons in the paraventricular nucleus of the hypothalamus secrete corticotropin-releasing hormone (CRH), which in turn releases adrenocorticotropic hormone (ACTH) from the anterior pituitary gland. ACTH enters the blood to stimulate the adrenal gland and regulate the release of glucocorticoids (cortisol) from the adrenal cortex (Herman et al., 2005; Nath et al., 2021). The stimuli also extend to the different brain areas, including the limbic system and other emotion centers, where they also modulate the release/level of norepinephrine (NE), dopamine, and 5-hydroxytryptamine (Hirschfeld, 2000). The hippocampus (HC) and prefrontal cortex (PFC) have also been found to play a critical role in mood disorders (Kosten et al., 2008). Antidepressants correct neuronal arborization in the HC, as suggested by the neurogenesis hypothesis of depression (Jacobs et al., 2000). At present, limited approaches with unavoidable adverse effects are available to treat/cure stress and depression.

Probiotics play an important role in acting as alternative medicines/drugs, filling the gap in the therapeutics of different psychopathologies and contributing to the pharmacological response with fewer adverse effects. First used formally by Lilly and Stillwell in 1965, the term probiotics is derived from the Greek word “pro bios,” meaning “for life” (Lilly and Stillwell, 1965; World Health Organization, 2002). The World Health Organization defined probiotics as “*Live microorganisms which when administered in adequate amount confer a health benefit on the host*” (World Health Organization, 2002; Morelli and Capurso, 2012). Probiotics include bacteria and yeast most commonly present in food (fermented milk) or presented as functional food/drugs. Different species of bacteria and yeast are commercially available all over the world (available in Pakistan: Enterogermina^®^, Newflora^®^, Enflor^®^, Ospor^®^, Amybact^®^, etc.), widely recognized as antidiarrheal and able to correct normal flora of the gastrointestinal tract. Probiotics may act through direct interaction in gastrointestinal tract disorders, e.g., colorectal cancer, irritable bowel syndrome, ulcerative colitis, and Crohn’s disease (Shanahan, 2011), or indirectly by modification of the immune system or vagus nerve stimulation (Mörkl et al., 2020). Probiotics are gaining more interest for their use in treating issues other than gastrointestinal tract diseases (Sun and Chang, 2014; Huang et al., 2016). The exact mechanisms of probiotics are still uncertain. Neurobiologists are taking more interest in exploring the mechanisms of probiotics in treating neurological disorders (Paré and Glavin, 1986; Foster and McVey Neufeld, 2013). Evidence from the literature has shown that the gastrointestinal tract’s microbiota influences the development and ramification of the brain (Huang et al., 2016). Normal flora of the gastrointestinal tract can affect behavioral functions. Bidirectional talk of the brain and the gut has long been recognized. Pathways that establish such communications are composed of the enteric nervous system in the gastrointestinal tract, immune system, autonomic nervous system, and neuroendocrine system. Neuroscientists are now noticing novel reports that highlight the “bottom-up” influence of microbes. In search of new therapeutic strategies to cure various neurological disorders and stressful conditions, probiotics are gaining attention because of their potential (Huang et al., 2016) in combating psychological disorders effectively. In the current study, the effects of locally produced *Lactobacillus fermentum* NMCC-14 (non-spore former) and commercially available spores of *Bacillus clausii* (Enterogermina^®^) were determined for their potential as curative/therapeutic agents in stress.

## 2 Materials and Methods

### 2.1 Animals

Albino C57BL/6J mice (male) weighing 23 ± 3 g, age 6–8 weeks, were purchased from the National Institute of Health, Islamabad, Pakistan, and transferred for initial acclimatization of 5 days to the animal house facility in the Department of Pharmacy, Quaid-i-Azam University, Islamabad, Pakistan. The temperature was maintained at 25 ± 2°C under 12/12 hours dark–light cycle. All experiments were performed according to the “guidelines and principles of laboratory animals” provided by the Bioethical Committee of Quaid-i-Azam University, Islamabad, with approval number **#BEC-FBS-QAU2021-266**.

Animals were randomly divided into seven groups (Table 1), each having five mice/experiment. Group I served as the normal group, where animals were allowed free access to food and autoclaved water without any probiotic treatment or stress induction. Group II acted as the negative control and included animals with free access to food and autoclaved water, followed by the restraint-stress procedure for 120 min/day in the early morning (Table 1). Group III was a positive group, and animals were administered fluoxetine (10 mg/kg/day, intraperitoneally (i.p)). Freshly prepared *Lactobacillus fermentum* NMCC-14 and *Bacillus clausii* at a dose of 10^10^ colony-forming unit (CFU)/ml/day were administered orally to the animals in Groups IV and V, respectively, followed by the restraint-stress procedure for 120 min. Animals in groups VI and VII were treated simply with freshly prepared *Lactobacillus fermentum* NMCC-14 (10^10^ CFU/ml/day) and *Bacillus clausii* (10^10^ CFU/ml/day) without any induction of stress. All treatments were continued for 7 days to evaluate the acute effect and 14 days to evaluate the subacute effect.

**TABLE 1:** Animal groups, probiotic treatment schedule, and behavioral test details.

Experimental set	Group (*n* = 5/group/experiment)	Drug/Probiotic treatment and behavioral studies schedule
Normal	I	No stress and probiotic treatment, Behavioral tests (EPM: acute, day 4; subacute, day 11; LDB: acute, day 5; subacute, day 12; OFT: acute, day 6; subacute, day 13)
Negative	II	Stress induced for 120 min/day, Behavioral tests performed 24 h after last stress induction, Behavioral tests (EPM: acute, day 4; subacute, day 11; LDB: acute, day 5; subacute, day 12; OFT: acute, day 6; subacute, day 13)
Positive	III	Fluoxetine 10 mg/kg in NS (i.p) + stress induced for 120 min/day, Behavioral tests performed 24 h after last stress induction, Behavioral tests (EPM: acute, day 4; subacute, day 11; LDB: acute, day 5; subacute, day 12; OFT: acute, day 6; subacute, day 13)
*Lactobacillus fermentum* NMCC-14 + Stress (LF-S)	IV	10^10^ CFU/day (p.o) + stress induced for 120 min/day, Behavioral tests performed 24 h after last stress induction (EPM: acute, day 4; subacute, day 11; LDB: acute, day 5; subacute, day 12; OFT: acute, day 6; subacute, day 13)
*Bacillus clausii* + Stress (BC-S)	V	10^10^ CFU/day (p.o) + stress induced for 120 min/day, Behavioral tests performed 24 h after last stress induction (EPM: acute, day 4; subacute, day 11; LDB: acute, day 5; subacute, day 12; OFT: acute, day 6; subacute, day 13)
*Lactobacillus fermentum* NMCC-14 (LF)	VI	10^10^ CFU/day (p.o), Behavioral tests performed (EPM: acute, day 4; subacute, day 11; LDB: acute, day 5; subacute, day 12; OFT: acute, day 6; subacute, day 13)
*Bacillus clausii* (BC)	VII	10^10^ CFU/day (p.o), Behavioral tests performed (EPM: acute, day 4; subacute, day 11; LDB: acute, day 5; subacute, day 12; OFT: acute, day 6; subacute, day 13)

Abbreviations: EPM, elevated plus maze; LDB, light dark box; OFT, open field test; i.p, intraperitoneal; p.o, per oral; NS, normal saline.

### 2.2 Probiotics

Spores of *Bacillus clausii* by Sanofi, 11 Viale Europa, Origgio, were purchased from the local pharmacy, available with the trade name Enterogermina^®^ (Batch Number: X1009, Reg. No. in Pakistan: 095289). *Lactobacillus fermentum* NMCC-14 (Accession number: MK611941.1, 586 bp, linear deoxyribonucleic acid (DNA)) (Ghazanfar et al., 2019), isolated from the milk of “Nilli Ravi Buffalo,” was provided by the probiotics lab, National Institute for Genomics and Advanced Biotechnology, *National Agricultural Research Centre*, Islamabad, Pakistan.

### 2.3 Chemicals

Chloroform, ethanol, formalin, orthophosphoric acid, 2-propanol, phosphate buffer—tween (Lot. N0. 00352, Part Number: 00501, Agdia, United States), dopamine hydrochloride (Lot No. BCBS3110V, Sigma Aldrich, Germany), nor-adrenaline (Alfa Aesar, Lot No. 1017520), fluoxetine (gift from Ferozson Laboratories, Nowshera, Pakistan), ACTH enzyme-linked immunoassay (ELISA) kit (Catalog No E026Mo, Bioassay Technology Laboratory, Shanghai, China) and cortisol ELISA kit (Catalog No. MBS269130, MyBioSource, Inc., San Diego, United States), TRIzol™ reagent (Catalog Number 15596018, Invitrogen ™, ThermoFisher Scientific, United States), Onescript cDNA synthesis kit (Catalog No. G236, Applied Biological Materials), and Maxima SYBER^®^ Green/ROX qPCR Master Mix (Lot No. 00737730 and 00720013, Thermoscientific, Lithuania) were used.

### 2.4 Stress Induction

Restraint or immobilization is a widely used and accepted model to study stress and depression in animals (Paré and Glavin, 1986; Liang et al., 2015; Seewoo et al., 2022). Restrainers (polyvinyl chloride, 50 ml syringe ventilated from the front by producing small vents) were used to induce stress in mice through previously reported protocols (Paré and Glavin, 1986; Zafir and Banu, 2009). After the administration of the probiotics (10^10^ CFU/ml/day, p.o) and fluoxetine (10 mg/kg/day, i.p), animals were locked static inside the syringe barrel by pulling the plunger toward the animal except leaving space for cervical movement in the front of the restrainer and placed for 120 min on an opaque surface. Mice were then removed from restrainers and returned to their cages for 5 min. Each animal was then evaluated for behavioral changes by using elevated plus maze (EPM), open field (OF), and dark light box (LDB) tests. All behavioral trials conducted were video recorded using a top-fitted camera system (iPhone 7). Parameter details for results were extracted from the videos for evaluation. All tests were performed on a separate day (EPM: acute on day 4 and subacute on day11, light dark box/dark light box (LDB): acute on day 5 and subacute on day 12, and OFT: acute on day 6 subacute on day 13) to avoid apparatus-induced stress in the mice.

### 2.5 Behavioral Tests

#### 2.5.1 Elevated Plus Maze

The EPM test has been extensively used to evaluate rodent behavior in stress, anxiety, and depression. EPM is a plus sign (+)-shaped wooden apparatus that consists of 2 open arms (length and width: 15 cm × 5 cm) and 2 closed arms (length, width, and height: 15 cm × 5 cm × 20 cm). Experiments were conducted through well-established protocols by Walf and Frye (2007), with slight modifications. EPM was adjusted 50 cm above the ground. After 120 min of restraining and 5 min of stay in the cage, on day 4 (for acute) and day 11 (for subacute), each animal of every group (Table 1) was put in the center of the EPM with its head facing one of the open arms. The animal was then allowed to explore the apparatus for 5 min, and time spent in the open and closed arms was used to assess stress behavior. All experiments were video recorded by the top-fitted camera.

#### 2.5.2 Dark Light Box

A DLB was used, following established protocols to measure stress behavior in mice (Bourin and Hascoët, 2003). The apparatus was composed of a wooden box divided into two chambers, one light compartment and one dark compartment. Animals from all groups (Table 1) were assessed for stress behavior after 120 min of restraining and 5 min of intermediate stay in the cage, on day 5 for acute and day 12 for subacute effects. Each animal was placed in the light compartment with its head facing the wall of the chamber. Time spent in the light and dark compartments (parameters for stress evaluation) was video recorded for 5 min using the top-fitted camera.

#### 2.5.3 Open Field Test

The open field test (OFT) assesses the locomotor activity and exploratory drive in rodents (Gould et al., 2009). The apparatus consists of square chambers (length, width, and height: 72 cm × 72 cm × 15 cm) divided into small squares (4 × 4) having an area of 18 cm × 18 cm. Each restrained (120 min) animal (Table 1) was kept in the center of the field on day 6 (for acute) and day 13 (for subacute) and allowed to explore the field for 5 min. Movable time and time spent in the center of the paradigm were recorded to evaluate the stress behavior using the top-fitted camera.

### 2.6 Serum Cortisol and Adrenocorticotropic Hormone Level by Enzyme-Linked Immunoassay

Blood samples were collected by cardiac puncture and transferred to a collecting tube. Serum was then extracted/separated by centrifugation for 15–20 min at 4°C and 2,000–3,000 rpm. Serum cortisol and ACTH levels were determined by ELISA according to the manufacturers’ instructions using an ACTH ELISA kit (Catalog No. E026Mo, Bioassay Technology Laboratory, Shanghai, China) and a cortisol ELISA kit (Catalog No. MBS269130, MyBioSource, Inc., San Diego, United States) respectively.

### 2.7 Histological Examination of Hippocampus

Animals were euthanized by cervical dislocation 24 h after the last restraining and activity in the behavioral paradigm. Brains removed were fixed in 4% paraformaldehyde and embedded in paraffin. Brains were then cut into a 5-µm thick section using a rotary microtome (Leica Biosystems). Hematoxylin and eosin (H&E) staining of each section was performed, and slides were developed and examined under an optical light microscope (Olympus. Model: CX41RF, Olympus Corporation, Tokyo, Japan).

### 2.8 Quantification of Monoamine Levels in Prefrontal Cortex and Hippocampus

The brain was dissected on an ice-cold Petri dish. Parts of the brains, HC, and PFC were removed and preserved in prechilled phosphate-buffered saline (PBS) (pH: 3.5, ingredients: 6.8 g potassium dihydrogen phosphate dissolved in 1000 ml of distilled water, and orthophosphoric acid was used to adjust the pH). Samples were then homogenized and centrifuged for 15–20 min at 4°C and 15,000 rpm. Supernatant collected was then filtered and used to detect the level of neurotransmitters in it. The mobile phase was composed of PBS and methanol (3:1, respectively). The column (material: 5 µm C18, dimensions: 4.6 × 250 mm, P/N 227-30017-08, S/N 17L05417) attached to high-performance liquid chromatography (HPLC, Shimadzu, detector: SPD-20AV, degasser unit: DGU-20ASR, and pump: LC-20AT) was adjusted to a wavelength of 270 nm and a flow rate of 1.5 ml to detect the neurotransmitter levels in the PFC and HC of mice. Next, 20 µl of the tissue sample prepared in PBS buffer was loaded to detect dopamine, serotonin, and NE levels in the PFC and HC. Standard solutions of dopamine, serotonin, and NE serially diluted (1 × 10^9^ ng/L, 5 × 10^8^ ng/L, 2.5 × 10^8^ ng/L, 1.25 × 10^8^ ng/L, and 0 ng/L) were first analyzed to confirm the retention time of each neurotransmitter (serotonin: 9 min, dopamine: 3.4 min, and NE: 1.9 min). Various neurotransmitter levels were analyzed using HPLC (LC-20AT Shimadzu, Kyoto, Japan). The concentration in each sample was calculated by the operating software LabSolution Lite (2008) provided by Shimadzu Corporation, Japan.

### 2.9 Quantitative Real-Time Polymerase Chain Reaction to Determine mRNA Expression of Dopamine Receptors (D_1_ and D_2_) and Synaptophysin

Left and right HC and PFC were removed from the brain on an inverted precooled Petri dish kept on ice. Both isolated HC and PFC were used to extract total RNA using TRIzol™ reagent (Catalog Number: 15596018, Invitrogen™, ThermoFisher Scientific, United States) according to the manufacturer’s protocols. After adjusting the concentration, RNA samples were converted to complementary DNA (cDNA) using a Onescript cDNA synthesis kit (catalog number: G236, Applied Biological Materials). cDNA of each sample was synthesized according to the provided protocols by the manufacturer. Samples were then used in qPCR for the measurement of messenger ribonucleic acid (mRNA) expression of glyceraldehyde 3-phosphate dehydrogenase (GADPH, housekeeping gene, primer used: forward 5′–3′: ACT​CCA​CTC​ACG​GCA​AAT​TCA; reverse 3′–5′: TCT​CGC​TCC​TGG​AAG​ATG​GT) (Allais et al., 2007), dopamine receptor subtypes, i.e., dopamine receptor 1 (D_1_, primer used: forward 5′–3′: AAC​TGT​ATG​GTG​CCC​TTC​TGT​GG; reverse 3′–5′: CAT​TCG​TAG​TTG​TTG​TTG​CCC​CG) (Zhu et al., 2007) and dopamine receptor 2 (D_2_, primer used: forward 5′–3′: CAC​TCC​GCC​ACT​TCT​TGA​CAT​ACA; reverse 3′–5′: TCT​CCT​CCG​ACA​CCT​ACC​CCG​A) (Gould et al., 2009), and synaptophysin (primer used: forward 5′–3′: AGA​CAG​GCA​GGT​GAA​GAG​GA; reverse 3′–5′: TTG​GCT​CTT​CCC​AGG​TTA​TG) (Allais et al., 2007). Maxima SYBER^®^ Green/ROX qPCR Master Mix (Lot Number: 00737730 and 00720013, Thermoscientific, Lithuania) was used in Applied Biosystem-installed with StepOne™ software Real-Time PCR System (Version 2.3, Life Technologies Corporation) for the relative quantification of each sample; then, 2^−∆∆CT^ was calculated after analyzing for expression level. Fold expression of dopamine receptor subtypes (D_1_ and D_2_) and synaptophysin (normalized with GADPH) was then expressed as fold change related to the normal (Table 1).

### 2.10 Statistical Analysis

Experimental data obtained from all groups are presented as mean ± standard error to the mean (SEM). Graphs were designed using Origin Lab 6.0 (United States). Statistical assessment was performed using two-way analysis of variance (ANOVA) to determine the significance of the difference (*p* ≤ 0.05) through IBM^®^ SPSS^®^ Statistics, version 25.

## 3 Results

### 3.1 Probiotics Ameliorate Stress Behaviors in Mice

Behavioral changes are primary indicators and can be observed in humans and animals to evaluate stress and depression. In animals, stress induction techniques vary from species to species. The restraint stress technique in mice is a well-reported method to observe the therapeutic effects of different drugs through various behavioral paradigms and other molecular and hormonal changes. The EPM, DLB, and OF tests are known paradigms used to evaluate the stress behavior of mice.

#### 3.1.1 Elevated Plus Maze

**FIGURE 1:**
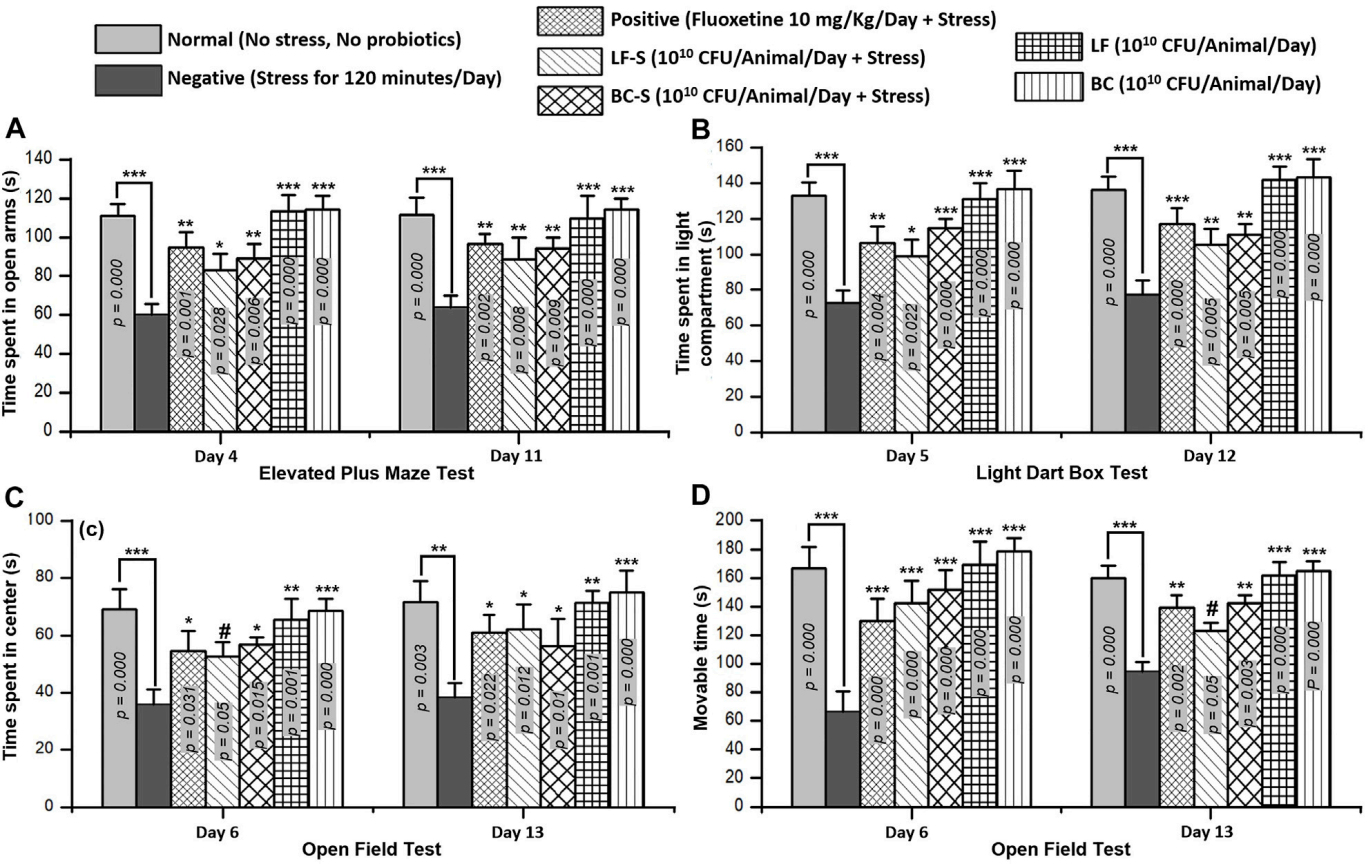
Effects of *Lactobacillus fermentum* NMCC-14 (10^10^ CFU/day, p.o) and *Bacillus clausii* (10^10^ CFU/day, p.o) in restraint-stressed and normal mice in **(A)** elevated plus maze (EPM) test, **(B)** light dark box (LDB) test, and **(C,D)** open field test (OFT). Compared to the negative group, *Lactobacillus fermentum* NMCC-14 (10^10^ CFU/day, p.o) and *Bacillus clausii* (10^10^ CFU/day, p.o) significantly (*n* = 5/group) increased the time spent in the open arms of EPM and the time spent in the light compartment of LDB as well as the movable time and time spent in the center of OFT in acute and subacute restraint-stressed mice. Compared to the normal group, no significant (*p* < 0.05) change in the animals’ behavior in groups BC and LF occurred. Values expressed are mean ± standard error to the mean (SEM). Note that ^*#*^ *p* = 0.05, *p < 0.05, **p < 0.01, and ***p < 0.001 versus the negative group by applying two-way ANOVA.

Time spent in the open arms of EPM is a parameter to evaluate the stress level in mice. Compared with the normal group (*n* = 5, Table 1), stress was induced in the negative group (*n* = 5) and time spent in the open arms was reduced significantly (*p* < 0.001, *n* = 5/group) both in acute and subacute restraint-stressed mice (Figure 1A). Fluoxetine (10 mg/kg, i.p), acting as a positive control (Table 1), suppressed stress behavior and significantly (*p* < 0.05, *n* = 5) increased the time spent in the open arms of the EPM in acute and subacute restraint-stressed mice (Figure 1A). Administration of *Lactobacillus fermentum* NMCC-14 (10^10^ CFU/day, p.o) and *Bacillus clausii* (10^10^ CFU/day, p.o) in Group VI (LF-S) and V (BC-S) animals (Table 1) also significantly (*p* < 0.05, *n* = 5) increased the time spent in the open arms of EPM in acute and subacute restraint-stressed mice in comparison to the negative group. Compared to the normal group (Group I), *Lactobacillus fermentum* NMCC-14 (10^10^ CFU/day, p.o) and *Bacillus clausii* (10^10^ CFU/day, p.o) showed no significant difference (*p* < 0.05, *n* = 5) in LF (Group VI) and BC (Group VII) mice, both in acute and subacute probiotics treatments (Figure 1A). In comparison, *Bacillus clausii* showed more positive effects than *Lactobacillus fermentum* NMCC-14 in the EPM experiment.

#### 3.1.2 Light Dark Box

Time spent in the light compartment indicates normal behavior, while time spent in the dark chamber represents stress behavior of the animal. Oral administration of *Lactobacillus fermentum* NMCC-14 (10^10^ CFU/day) and *Bacillus clausii* (10^10^ CFU/day) suppressed stress behavior in the LF-S and BC-S groups (Table 1) and significantly (*p* < 0.05, *n* = 5) increased the time spent in the light compartment in acute and subacute restraint-stressed mice (Figure 1B). Fluoxetine (10 mg/kg, i.p) also significantly (*p* < 0.01, *n* = 5) increased the time spent in the light compartment both in acute and subacute restraint-stressed mice (Figure 1B). Compared to the normal group, no significant (*p* < 0.05, *n* = 5) changes were observed in the time spent in the light compartment by administration of *Lactobacillus fermentum* NMCC-14 (10^10^ CFU/day, p.o) and *Bacillus clausii* (10^10^ CFU/day, p.o) in the LF and BC groups (Table 1) in acute and subacute probiotics-fed mice. Results from all groups showed more stress-suppressing effects of *Bacillus clausii* than *Lactobacillus fermentum* NMCC-14 in the LDB test.

#### 3.1.3 Open Field Test

Movable and immovable time and time spent in the central arena of the apparatus are parameters used to evaluate stress behavior in mice. Compared to the negative group (*n* = 5), *Bacillus clausii* (10^10^ CFU/day, p.o) and *Lactobacillus fermentum* NMCC-14 (10^10^ CFU/day, p.o) significantly (*p* ≤ 0.05, *n* = 5) increased the movable time in the LF-S and BC-S animal groups (Table 1) in acute and subacute restraint-stressed mice (Figure 1C). Time spent in the central arena was also increased significantly (*p* ≤ 0.05, *n* = 5) by the oral administration of *Bacillus clausii* (10^10^ CFU/day) and *Lactobacillus fermentum* NMCC-14 (10^10^ CFU/day) in the LF-S and BC-S group animals, both in acute and subacute restraint-stressed mice (Figure 1D). Fluoxetine (10 mg/kg, i.p, positive group) (*n* = 5, Table 1) also significantly (*p* < 0.05, *n* = 5) increased the movable time and time spent in the center of the OF both in acute and subacute restraint-stressed mice (Figures 1C and D). Compared to the normal group, no significant (*p* < 0.05, *n* = 5) changes were observed by acute and subacute administration of *Bacillus clausii* (10^10^ CFU/day, p.o) and *Lactobacillus fermentum* NMCC-14 (10^10^ CFU/day, p.o) in animals in the LF and BC groups (Table 1) in movable time and time spent in the center of the open field. Results from OFT showed that treatment with *Bacillus clausii* produces more positive effects than treatment with *Lactobacillus fermentum* NMCC-14, except in movable time in subacute conditions, where the effects of *Lactobacillus fermentum* NMCC-14 were more prominent than those of *Bacillus clausii*.

### 3.2 Serum Cortisol and Adrenocorticotropic Hormone Level

**FIGURE 2:**
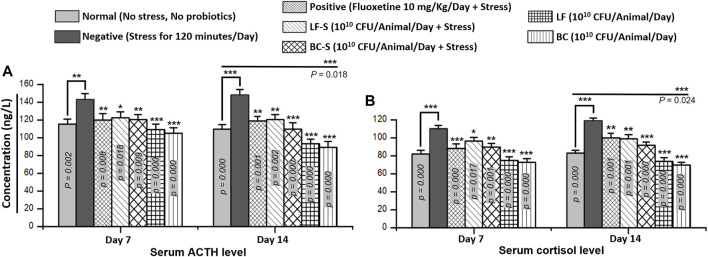
Effects of *Lactobacillus fermentum* NMCC-14 (10^10^ CFU/day, p.o) and *Bacillus clausii* (10^10^ CFU/day, p.o) on serum **(A)** adrenocorticotropic hormone (ACTH) and **(B)** cortisol levels. Compared to the negative group, *Lactobacillus fermentum* NMCC-14 (10^10^ CFU/day, p.o) and *Bacillus clausii* (10^10^ CFU/day, p.o) significantly (*n* = 5/group) reduced the ACTH and cortisol levels in the LF-S and BC-S groups. Compared to the normal group, BC significantly decreased (day 14: ACTH, *p* = 0.018; cortisol, *p =* 0.024) the ACTH level in the BC group animals on day 14 and no significant (*p* < 0.05) change in the ACTH and cortisol levels was observed both in the LF and other BC group animals. Values expressed are mean ± standard error to the mean (SEM). Note that ^*#*^ *p* = 0.05, *p < 0.05, **p < 0.01, and ***p < 0.001 versus the negative group by applying two-way ANOVA.

Cortisol, the main glucocorticoid from the adrenal gland, plays a central role in glucose metabolism and in the response of the body to stress. The production of adrenal cortisol is regulated by the pituitary hormone adrenocorticotropic hormone, synthesized in response to hypothalamic corticotrophin-releasing hormone. Compared to the negative group (*n* = 5), *Bacillus clausii* (10^10^ CFU/day, p.o) and *Lactobacillus fermentum* NMCC-14 (10^10^ CFU/day, p.o) treatment significantly (*p* < 0.05, *n* = 5) reduced the adrenocorticotropic hormone and cortisol levels in Group IV (LF-S) and Group V (BC-S) animals (Table 1), both in acute and subacute restraint-stressed mice (Figures 2A and B). Fluoxetine (10 mg/kg, i.p) also significantly (*p* < 0.05, *n* = 5) reduced the adrenocorticotropic hormone and cortisol levels in acute and subacute restraint-stressed mice (Figures 2A and B). Compared to the normal group, except ACTH and cortisol levels in mice in group VII (BC) on day 14, no significant (*p* < 0.05, *n* = 5) change in serum ACTH and cortisol levels in Group VI (LF) and VII (BC) was observed, both with acute and subacute administration of *Bacillus clausii* (10^10^ CFU/day, p.o) and *Lactobacillus fermentum* NMCC-14 (10^10^ CFU/day, p.o) (Table 1; Figures 2A andB). All results of ACTH and cortisol levels in the blood serum of mice showed that *Bacillus clausii* produced a greater decrease in ACTH and cortisol levels than *Lactobacillus fermentum* NMCC-14 and will be more effective to treat stress conditions.

### 3.3 *Lactobacillus fermentum* NMCC-14 and *Bacillus clausii* Improve Neurodegeneration in the Hippocampus

**FIGURE 3:**
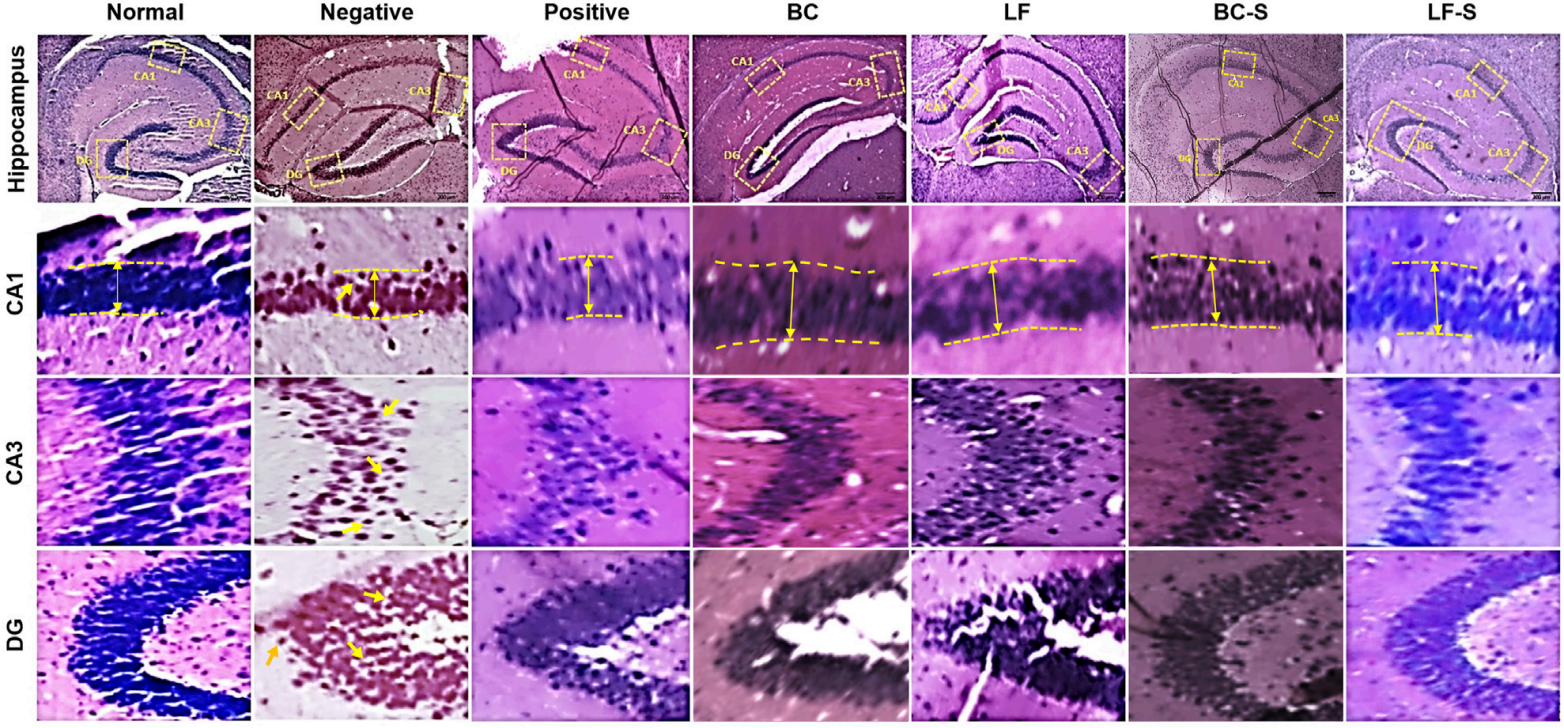
Effects of *Lactobacillus fermentum* NMCC-14 (10^10^ CFU/day, p.o) and *Bacillus clausii* (10^10^ CFU/day, p.o) on the hippocampus CA1, CA3, and DG of acute restraint-stressed and normal mice. *Lactobacillus fermentum* NMCC-14 (10^10^ CFU/day, p.o) and *Bacillus clausii* (10^10^ CFU/day, p.o) improved stress-induced neurodegenerative changes in the hippocampus CA1, CA3, and DG regions (H&E staining; magnification ×10, scale bar: 200 µm).

**FIGURE 4:**
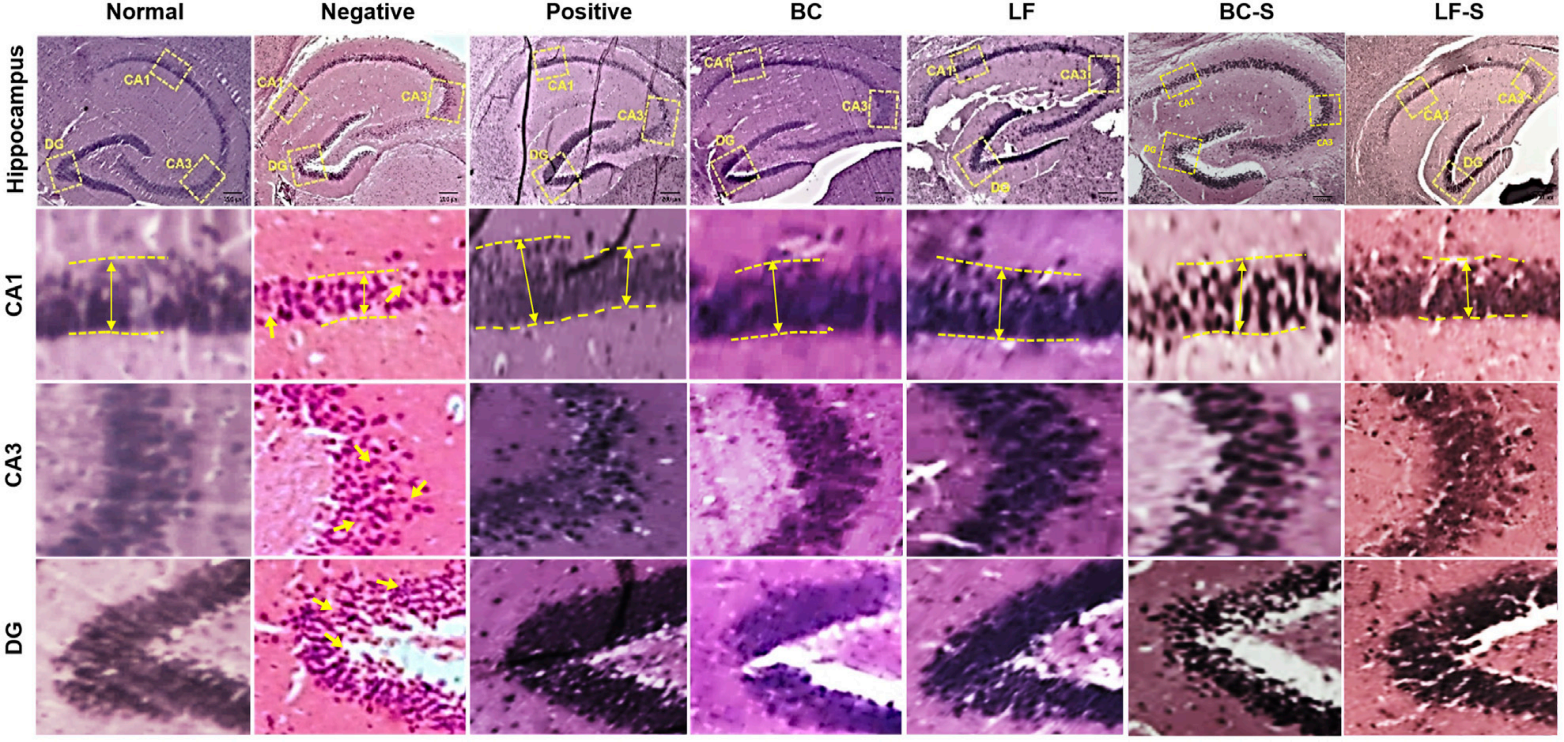
Effects of *Lactobacillus fermentum* NMCC-14 (10^10^ CFU/day, p.o) and *Bacillus clausii* (10^10^ CFU/day, p.o) on the hippocampus CA1, CA3, and DG of subacute restraint-stressed and normal mice. *Lactobacillus fermentum* NMCC-14 (10^10^ CFU/day, p.o) and *Bacillus clausii* (10^10^ CFU/day, p.o) improved stress-induced neurodegenerative changes in the hippocampus CA1, CA3, and DG regions (H&E staining; magnification ×10, scale bar: 200 µm).

Neurodegeneration in the cornu ammonis (CA1 and CA3) and dentate gyrus (DG) of the HC was investigated for the effects of *Lactobacillus fermentum* NMCC-14 (10^10^ CFU/day, p.o) and *Bacillus clausii* (10^10^ CFU/day, p.o) in restraint-stressed and normal mice (Table 1; Figures 3 and 4). Histomorphological alterations showed neurodegeneration and pyknosis in acute and subacute restraint-stressed mice (negative group—Group II, Table 1 and Figures 3 and 4). Slides of the brains of negative group mice, both in acute and subacute restraint-stressed conditions, showed retraction of neuronal processes, condensed arborization of neurons toward the nucleus (pyknosis), and reduced width of CA1 of the HC, as shown in Figures 3 and 4. Animals in the normal, LF (Group VI), and BC (Group VII) showed no obvious abnormalities or degeneration of neurons of the CA1, CA3, and dentate gyrus of the HC (Figures 3 and 4). Orally administered *Bacillus clausii* (10^10^ CFU/day) and *Lactobacillus fermentum* NMCC-14 (10^10^ CFU/day) also resulted in improvement in the neuronal arborization in DG, CA1, and CA3 of the HC in the brains of restraint-stressed mice of Group IV and V as compared to negative group (Group II).

### 3.4 Effects of *Lactobacillus fermentum* NMCC-14 and *Bacillus clausii* on Monoamine Level in the Prefrontal Cortex and Hippocampus

**FIGURE 5:**
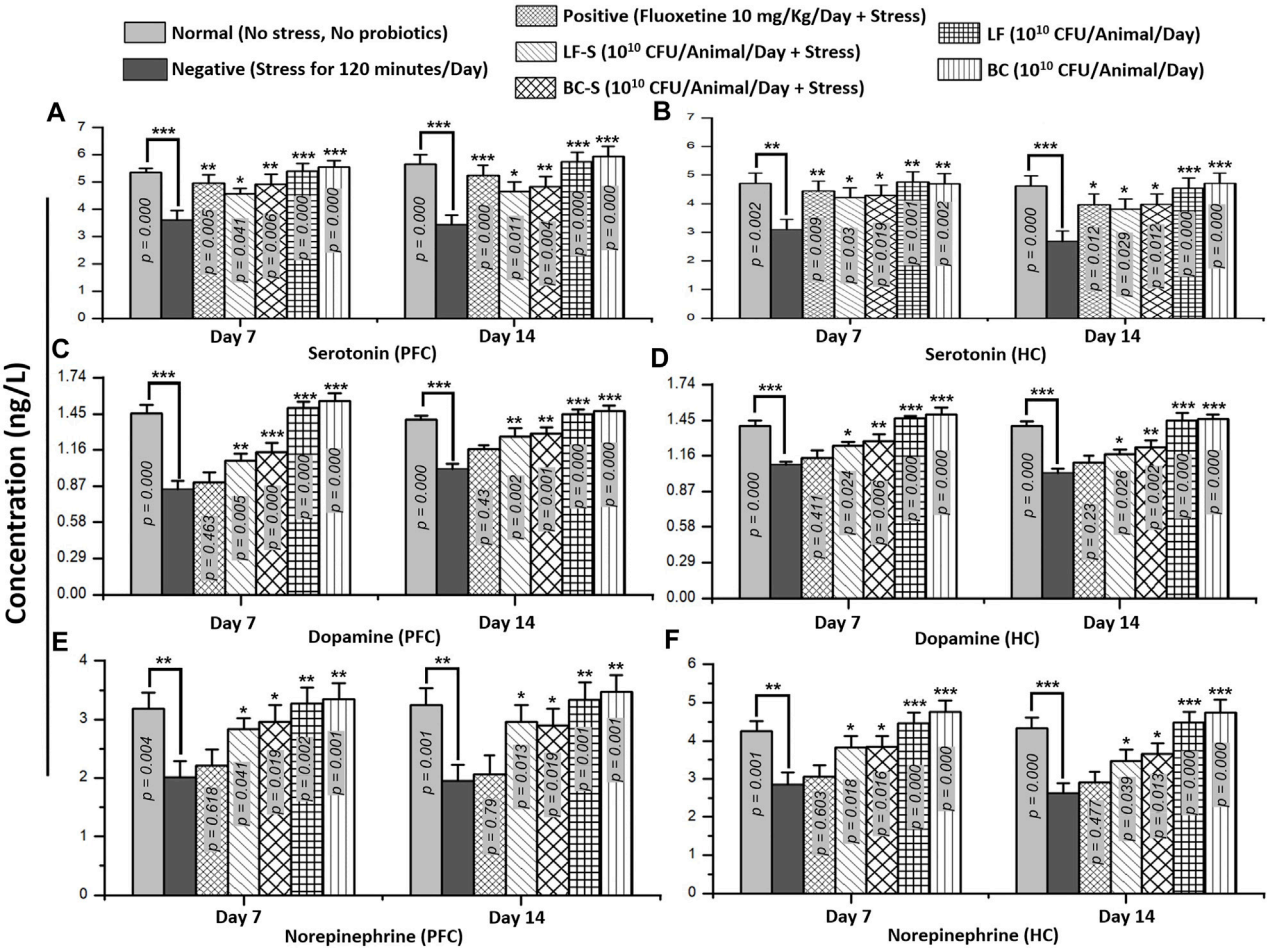
Effects of *Lactobacillus fermentum* NMCC-14 and *Bacillus clausii* on monoamine levels (serotonin, dopamine, and norepinephrine) in the prefrontal cortex and hippocampus of acute and subacute restraint-stressed and normal mice. Compared to the negative group (*n* = 5/group), a significant increase in PFC and HC monoamine levels—serotonin **(A,B)**, dopamine **(C,D)**, and norepinephrine **(E,F)**—was shown in LF-S and BC-S group restraint-stressed mice. Compared to the normal group (*n* = 5/group), no significant (*p* < 0.05) change was observed in monoamine levels—serotonin **(A,B)**, dopamine **(C,D)**, and norepinephrine **(E,F)**—in the PFC and HC of LF and BC group mice. Values expressed are mean ± standard error to the mean (SEM). Note that ^*#*^ *p* = 0.05, *p < 0.05, **p < 0.01, and ***p < 0.001 versus the negative group by applying two-way ANOVA.

Stress and depression are associated with monoamine levels in the synaptic cleft. An increase in the monoamine level at synapses improves mood disorders, as proposed in the “monoamine hypothesis” (Hirschfeld, 2000). Stressors with short bursts and less duration increase the monoamine level, but a decrease is observed with persistent and recurrent induction of stress in animals (Dishman, 1997). Compared to the negative group (Table 1), *Bacillus clausii* (10^10^ CFU/day, p.o) and *Lactobacillus fermentum* NMCC-14 (10^10^ CFU/day, p.o) significantly (*p* < 0.05, *n* = 5/group) increased the serotonin, dopamine, and NE levels in acute acute and subacute restraint-stressed mice (Figures 5A–F). Fluoxetine (10 mg/kg, i.p, positive group) also significantly (*n* = 5, *p* < 0.05) increased only the level both in acute and subacute restraint-stressed mice. The fluoxetine group (10 mg/kg, i.p) did not show any significant (*n* = 5, *p* < 0.05) increase versus the negative group in the level of dopamine and NE in acute acute and subacute restraint-stressed mice. Compared to the normal group animals (Table 1), no significant (*p* < 0.05, *n* = 5, Figures 5A–F) changes in the serotonin, dopamine, and NE levels were observed in the LF (Group VI) and BC (VII), both in acute acute and subacute restraint-stressed mice. In comparison, *Bacillus clausii* showed a greater increase in the monoamine level than *Lactobacillus fermentum* NMCC-14.

### 3.5 Effects of *Lactobacillus fermentum* NMCC-14 and *Bacillus clausii* on the mRNA Expression of Dopamine Receptors (D_1_ and D_2_) and Synaptophysin in the Prefrontal Cortex and Hippocampus

**FIGURE 6:**
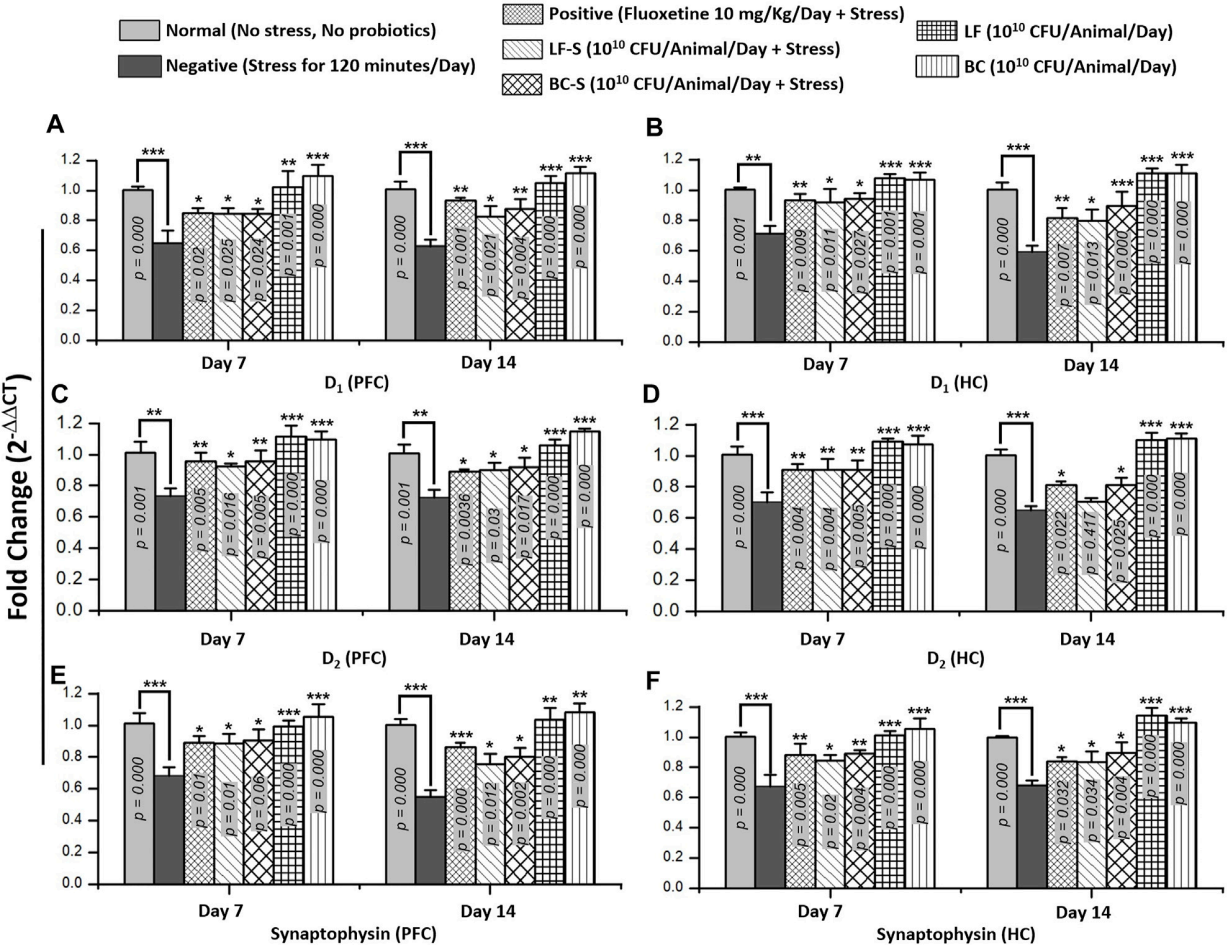
Effects of *Lactobacillus fermentum* NMCC-14 (10^10^ CFU/day, p.o) and *Bacillus clausii* (10^10^ CFU/day, p.o) on mRNA levels of dopamine receptors (D_1_ and D_2_) and synaptophysin in the prefrontal cortex and hippocampus of acute and subacute restraint-stressed and normal mice. Compared to the negative group, the mRNA expression of dopamine receptor subtypes (D_1_ and D_2_) and synaptophysin increased in the PFC **(A,C,E)** and HC **(B,D,F)** significantly (*n* = 5/group) by acute and subacute administration of *Lactobacillus fermentum* NMCC-14 and *Bacillus clausii* in LF-S (except D_2_ in the HC, day 14) and BC-S mice. Compared to the normal group, no significant (*p* < 0.05) change was observed in the mRNA expression of dopamine receptor subtypes (D_1_ and D_2_) and synaptophysin in the PFC **(A,C,E)** and HC **(B,D,F)** of BC and LF mice. Values expressed are mean ± standard error to the mean (SEM). Note that ^*#*^ *p* = 0.05, *p < 0.05, **p < 0.01, and ***p < 0.001 versus the negative group by applying two-way ANOVA.

Stress is a multifarious ailment in which changes in neurotransmission occur. Expression of different monoamine receptor subtypes, e.g., dopamine D_1_ and D_2_, and synaptic vesicle release-regulating proteins, e.g., synaptophysin, is altered under conditions of stress and depression. Compared to the negative group (except D_2_, HC in subacute condition), *Lactobacillus fermentum* NMCC-14 and *Bacillus clausii* treatment significantly (*p* < 0.05, *n* = 5) reversed the downregulation of mRNA expression of D_1_, D_2_, and synaptophysin in acute and sub-acute restraint-stressed mice (Figures 6A–F). Fluoxetine also significantly (*p* < 0.05, *n* = 5) upregulated stress-induced suppression of D_1_, D_2_, and synaptophysin mRNA expression in acute and subacute restraint-stressed mice (Figures 6A–F). In groups VI (LF) and VII (BC), the animals did not show significant (*p* < 0.05, *n* = 5) changes in the fold expression of D_1_, D_2_, and synaptophysin mRNA, both in acute and subacute probiotics fed mice, as compared to animals in the normal group. Results from dopamine receptor (D_1_ and D_2_) and synaptophysin expressions showed that *Bacillus clausii* treatment caused a greater increase in mRNA expression than *Lactobacillus fermentum* NMCC-14 treatment, both in acute and subacute restraint-stressed mice and LF and BC group animals.

## 4 Discussion

Stress is characterized by feeling overwhelmed and frustrated with a lack of interest in the things that once seemed interesting. Neurobiologists are exploring new entities to find the best, safest, and most definitive/targeted therapy for such a disorder. Probiotics are becoming prominent and gaining interest in molecular biology to unveil the possible mechanisms of action of microorganisms/microbiota in treating various neurological disorders. Studies mostly conducted on probiotics utilize chronic stress or the restraint-stressed model rather than any acute or subacute condition. Acute and subacute persistent stress mediate or promote depression and other complex psychopathologies. (Esch et al., 2002). Chronic pathologies can be prevented by correcting acute stress conditions early. For this reason, despite one time acute stressor condition, we focused on acute (7 days) and subacute (14 days) daily stress induction, which further can be considered a progressive or promoting factor to cause depression and other serious chronic neuropathological conditions. In this study, probiotic strains, *Lactobacillus fermentum* NMCC-14 and spores of *Bacillus clausii* (Enterogermina^®^), were evaluated for their role in treating acute and subacute restraint-stressed mice.

Our experimental results revealed that probiotics suppress symptoms of stress in acute and subacute restraint-stressed mice in behavioral paradigms, i.e., EPM, LDB, and OFT. Stress was reduced or suppressed by decreasing the serum cortisol and ACTH levels, increasing the monoamine (serotonin, dopamine, and NE) levels, and increasing the mRNA expression of dopamine (D_1_ and D_2_) receptors and synaptophysin. Moreover, enhancement in neurological arborization was also observed in the H&E slides on neurodegenerative effects and pyknosis caused by restraint stress in the mice HC.

Spores of *Bacillus clausii*, available with the trade name Enterogermina®, are registered as over-the-counter drugs and used as an antidiarrheal in humans. *Bacillus clausii*, spore-forming bacteria, has advantages over the *Lactobacillus* species as it is heat stable and can be stored at room temperature without any deleterious effects on its viability; it is also resistant to the acidic gastric environment. *Bacillus* species are extensively studied for their effects on dysbiosis and diarrhea. A study also showed that *Bacillus clausii* reduced the duration of respiratory tract infections in children (Marseglia et al., 2007). Shahgond et al. (2022) further reported that *Bacillus clausii* UBBC07 combined with *Lactobacillus plantarum* UBLP40 was effective in acute hepatic encephalopathy. A study conducted by Yunes and coworkers reported that *Bifidobacterium adolescentis* 150 and *Lactobacillus plantarum* 90sk showed a reduction in depressive behavior like that of fluoxetine in a force swimming test (Yunes et al., 2020). It is also reported that environmental enrichment suppresses stress behavior in an animal model and can greatly be approached to contribute to the treatment of mental disorders (Dandi et al., 2018). *Lactobacillus fermentum* has also been studied extensively for its role in treating many diseases, like inflammatory bowel disease (Bao et al., 2010), respiratory tract infections (Maldonado et al., 2012), and psychological abnormalities (Wang et al., 2015). In a similar manner, many studies have been conducted to test different species of *Lactobacilli* (*L. helveticus*, *L. rhamnosus*, *L. plantarum*, and *L. fermentum*) and *Bifidobacteria* (*B. longum* and *B. breve*) using EPM, LDB, OFT, fear conditioning, and step-down tests to evaluate stress (Bercik et al., 2010; Savignac et al., 2014; Liang et al., 2015; Wang et al., 2015; Liu WH et al., 2016; Liu YW et al., 2016) and depression (Desbonnet et al., 2008; Desbonnet et al., 2010; Bravo et al., 2011; Singh et al., 2012; Savignac et al., 2014; Liu WH et al., 2016; Liu YW et al., 2016) in animals. All these studies showed positive effects of probiotics in these behavioral paradigms, which greatly support the results of our study.

Sudo et al. (2004) demonstrated the elevation of ACTH and corticosterone levels in restraint-stressed mice. Another study showed that increased corticosterone levels were prevented by environmental enrichment in maternally separated stressed Wistar rat pups (Dandi et al., 2018). Fluoxetine was also found to normalize corticosterone serum levels (Wei et al., 2019). Neurotransmissions and their regulation contribute to all central nervous system physiology, including stress and depression. Like canonical gap junctions and mechanosensitive ion channels, synaptophysin is a transmembrane synaptic vesical release-regulating protein and marker for neuronal damage (Arthur and Stowell, 2007; Gudi et al., 2017; White and Stowell, 2021). Sze et al. (1997) observed a decrease in synaptophysin expression in Alzheimer’s disease. Thome et al. (2001) also reported decreased synaptophysin and synaptotagmin mRNA expression in stressed rats. Stress can ameliorate or have detrimental effects on the brain if it persists in the long term and causes neurodegeneration in the HC (Esch et al., 2002). A study by Dandi et al. (2018) revealed that environmental enrichment increased the expression of brain-derived neurotrophic factors and synaptophysin in stressed Wistar rat pups. Heat-killed and live *Lactobacillus paracasei* PS23 was also found to reverse the corticosterone-induced reduction in the serotonin and dopamine levels in mice (Wei et al., 2019). Desbonnet and his colleagues reported enhancement of the neurotransmitters NE, dopamine, and serotonin and reduced neurotransmitter metabolites, i.e., dihydroxyphenylacetic acid and 5-hydroxy indole acetic acid, in probiotic-treated Sprague Dawley rats (Desbonnet et al., 2008). The dopaminergic neuronal system plays an important role in mental disorders, and studies from the literature reported that the activation of dopamine (D1 and D2) receptor subtypes is necessary to reduce symptoms of stress behaviors (Kamei et al., 1995; Sim et al., 2013). Dopamine exerts stimulatory effects on HPA both through D_1_ and D_2_ receptors to provoke severe stress (Belda and Armario, 2009). A study conducted by Kamei and coworkers reported that the activation of both D_1_ and D_2_ receptors is necessary to attenuate fear-conditioned stress in mice (Kamei et al., 1995).

Humans harbor nearly 100 trillion bacteria in their gastrointestinal tract, which play an important role in maintaining normal health (Gill et al., 2006). *Bacillus*, including *Lactobacillus*, species are normal inhabitants of the human intestinal tract among the probiotics (Pereira et al., 2003). Probiotic use decreases several pathogenic gastrointestinal microorganisms, reduces bloating effects and flatulence, and improves bowel regularity. In humans, *Lactobacillus fermentum* NMCC-14 and *Bacillus clausii* as well as other species of gut microbiota can be used to reduce anxiety and to modulate the brain biochemical system. New insights are continuously emerging, and researchers strive to explore the human gut microbiota for a better alternative to the already available remedies or to provide new strategies to treat stress and other neurological disorders.

**FIGURE 7:**
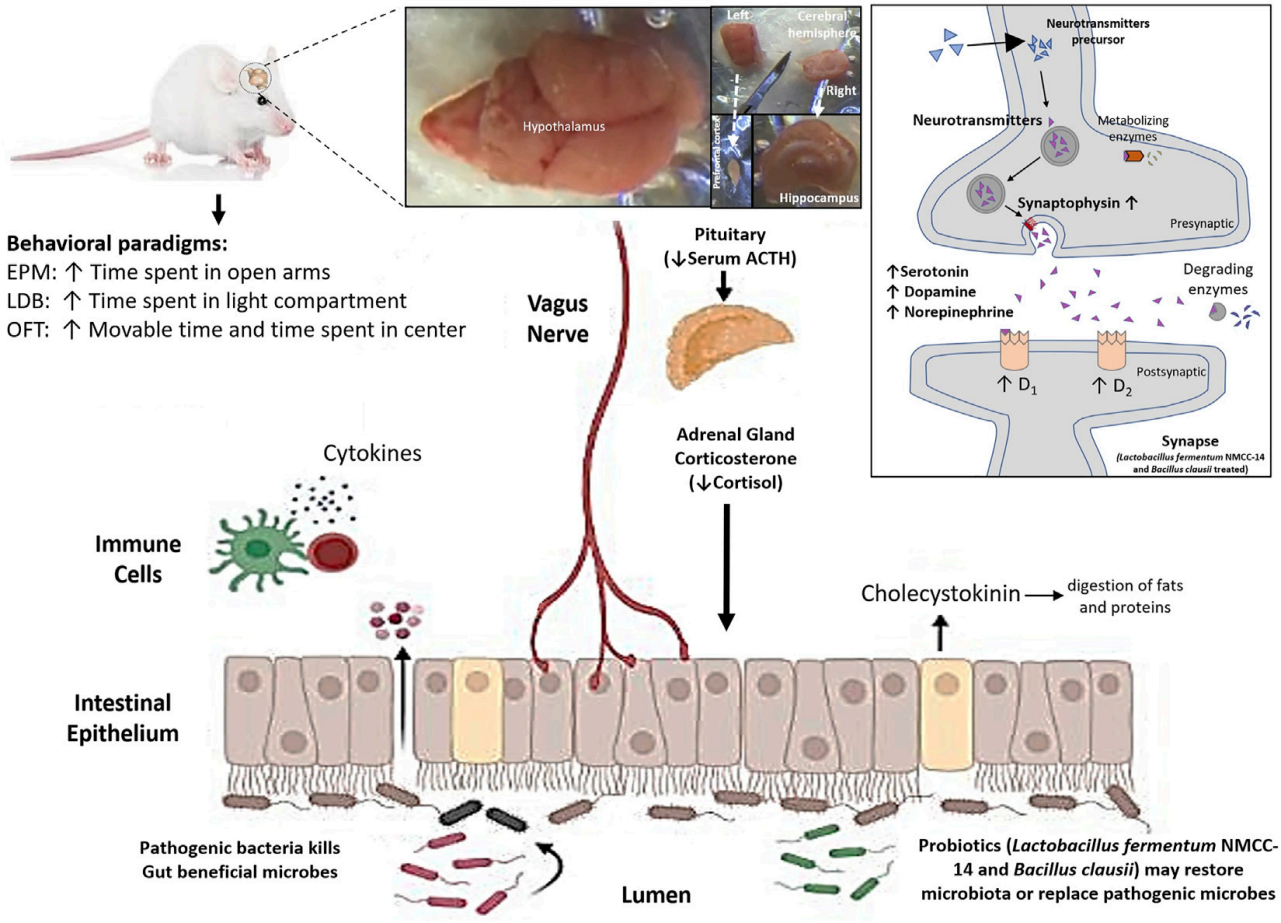
Effects of *Lactobacillus fermentum* NMCC-14 (10^10^ CFU/day, p.o) and *Bacillus clausii* (10^10^ CFU/day, p.o) through the gut–brain axis on monoamine levels (serotonin, dopamine, and norepinephrine), mRNA expression of synaptophysin and dopamine receptor subtypes (D_1_ and D_2_) in the hippocampus and prefrontal cortex of mice brains, and blood ACTH and cortisol levels in restraint-stressed and normal mice. Note that ↑ shows increase and ↓ shows decrease, and the abbreviations used are **EPM**, elevated plus maze, **LDB**, light dark box, and **OFT**, open field test.

The findings of these studies in the literature extend support in one way or another to our results, which further validate the role of probiotic use in stress and depression or any other neurological disorder. The mechanism of action is versatile for probiotics and not possible to define in a single way for any disease. Significant suppression of stress can be achieved by multimodal effects as indicated in our study. Probiotics may replace the pathogenic microbes, act as a digestive aid to improve nutritional contents, enhance the availability of more precursors of monoamines and finally to increase the level of serotonin, dopamine, and NE (Figure 7). Probiotics also upregulated dopamine receptor subtypes (D_1_ and D_2_) and synaptophysin mRNA expression and can also increase the cellular response and release of monoamines in the HC and PFC neurons. Central modulation of the brain then decreased blood ACTH and cortisol levels. The major effects may also have occurred by enhancing the immune system and vagal stimulation either directly from the brain or through the enteric nervous system (Figure 7). All these positive alterations significantly improved the stress behavior of mice in the LF and BC groups.

Terminologies like “psychobiotics” (Sharma et al., 2021) to treat neurological diseases and “pharmabiotics” (Lee et al., 2018; Sharma and Im, 2018), in general, are emerging to explore new insights into probiotics for their role in different neurological disorders. Our study’s evaluation of probiotics revealed that both spore-forming *Bacillus clausii* (Enterogermina^®^) and non-spore-forming *Lactobacillus fermentum* NMCC-14 strains significantly improved activity and performance and corrected biological functions in restraint-stressed mice. *Bacillus clausii* caused a greater improvement in stressed and normal animals *than Lactobacillus fermentum* NMCC-14. No stress-inducing effects by *Lactobacillus fermentum* and *Bacillus clausii* (groups VI and VII) were observed compared to the normal group, but these probiotics even enhanced the activity of mice. *Lactobacillus fermentum* NMCC-14 and *Bacillus clausii* (Enterogermina^®^) can be a safer and better single therapeutic entity or in combination with already available drugs to treat stress and other mental disorders.

## Data Availability

The datasets presented in this study can be found in online repositories. The names of the repository/repositories and accession number(s) can be found in the article/Supplementary Material.

## Abbreviations

ACTH, Adrenocorticotropic hormone CA, Cornu ammonis; CFU, Colony-forming unit; corticotrophin-releasing hormone, Corticotropin-releasing hormone; cDNA, Complementary DNA; DNA, Deoxyribonucleic acid; D_1_, Dopamine Receptor 1; D_2_, Dopamine Receptor 2; DG, Dentate gyrus; ELISA, Enzyme-linked immunoassay; EPM, Elevated plus maze; GADPH, Glyceraldehyde 3-phosphate dehydrogenase; HPLC, High-performance liquid chromatography; H&E, Hematoxylin and eosin; i.p, Intraperitoneal; LDB, light dark box/dark light box; mRNA, Messenger ribonucleic acid; NE, Norepinephrine; NS, Normal saline; OFT, Open field test; p.o, Per oral; qPCR, Quantitative polymerase chain reaction; vs., versus.
